# One master to rule them all

**DOI:** 10.7554/eLife.82873

**Published:** 2022-09-23

**Authors:** Pablo Barbeito, Francesc R Garcia-Gonzalo

**Affiliations:** 1 Departamento de Bioquímica, Facultad de Medicina, Universidad Autónoma de Madrid Madrid Spain; 2 Instituto de Investigaciones Biomédicas “Alberto Sols”, Consejo Superior de Investigaciones Científicas Madrid Spain; 3 CIBER de Enfermedades Raras, Instituto de Salud Carlos III Madrid Spain

**Keywords:** centriole, multiciliated, cilia, deuterosome, Plk4, Mouse

## Abstract

Multiciliated cells rely on the same master regulator as dividing cells to amplify the number of centrioles needed to generate the hair-like structures that coat their cell surface.

**Related research article** LoMastro GM, Drown CG, Maryniak AL, Jewett CE, Strong MA, Holland AJ. 2022. PLK4 drives centriole amplification and apical surface area expansion in multiciliated cells. *eLife* **11**:e80643. doi: 10.7554/eLife.80643.

The skeleton of filaments that gives animal and other eukaryotic cells their shape is organized in large part by two cylindrical structures called centrioles. Every cell cycle, the centrioles duplicate to form the mitotic spindle that separates the cell’s genetic material evenly between the daughter cells during cell division (Figure 1A). If the centrioles fail to replicate or too many are made, this can lead to diseases like cancer or microcephaly (Nigg and Holland, 2018).

**Figure 1.**
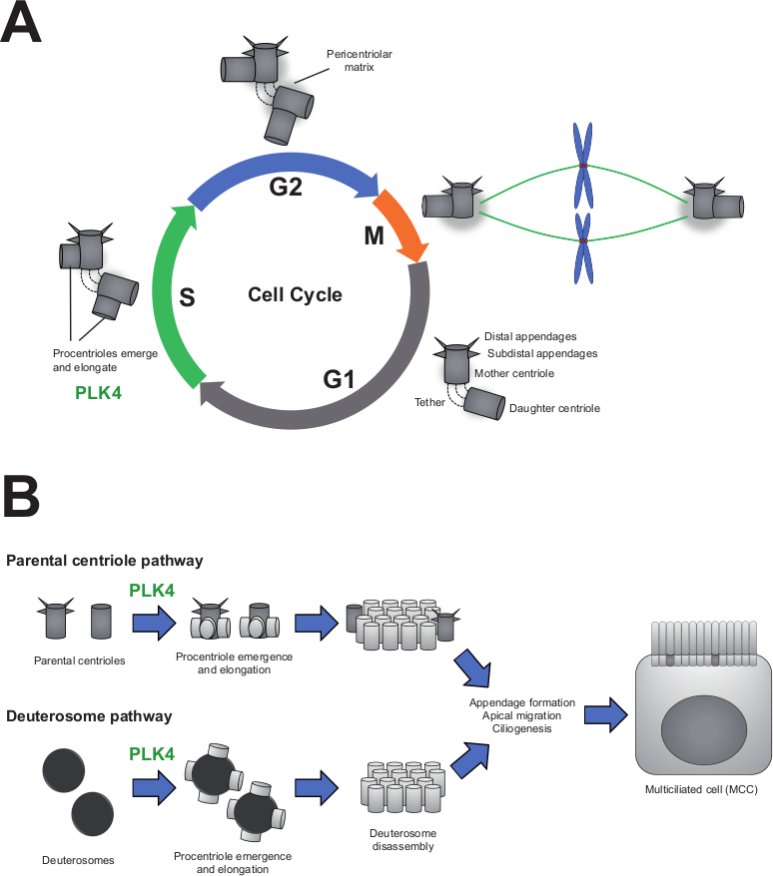
PLK4 is essential for centriole assembly in both cycling cells and differentiating multiciliated cells. (**A**) In the first phase of the cell cycle (known as G1), each cell contains two centrioles (grey cylinders): the mother centriole, which is fully mature and contains appendages, and the daughter centriole, which is younger and appendage-free. These centrioles are tethered together and surrounded by an amorphous mass of protein known as the pericentriolar matrix. As cells replicate their DNA (S-phase), each centriole duplicates to form new ‘procentrioles’ via a mechanism regulated by the enzyme PLK4. The procentrioles then elongate into fully formed centrioles. By the G2 phase of the cycle, cells have two pairs of centrioles, which form the mitotic spindle during mitosis (M-phase). The spindle then segregates the centrioles and the cell’s genetic material evenly among the daughter cells. (**B**) Multiciliated cells (MCCs) arise from progenitor cells that exit their last mitosis with two centrioles, as shown above. During differentiation, these parental centrioles are amplified via two distinct mechanisms: parental centrioles duplicating multiple times to produce new centrioles (top), or centrioles being built from scratch (bottom) using spheroidal, transitory organelles called deuterosomes. Eventually, all the centrioles mature and migrate to the apical surface where they dock to the membrane and form the cell’s cilia. In progenitor cells lacking the enzyme PLK4, neither of these pathways are functional and the cells are unable to differentiate into multiciliated cells.

Image credit: Pablo Barbeito and Francesc R. Garcia-Gonzalo.

Centrioles are also responsible for forming hair-like structures known as cilia, which protrude from the body of certain cells. Some epithelial cells have hundreds of cilia attached to their apical surface, which function like oars in a galley, collectively beating to displace extracellular fluid. These multiciliated cells can be found lining the surface of several organs, including the trachea, reproductive tissues, and the ventricular system in the brain. However, defects in these cells can lead to respiratory infections, infertility, and other cilia-associated disorders (Reiter and Leroux, 2017).

Multiciliated cells arise during development from epithelial progenitor cells that each contain a single centriole pair. To form the hundreds of cilia that will coat the apical surface, the progenitors increase their centriole content either by copying the original pair multiple times, or by building new centrioles in transitory structures called deuterosomes (Figure 1B; Nanjundappa et al., 2019).

During this amplification process (known as A-phase), the levels of an enzyme called PLK4 (short for Polo-like kinase 4) increase, and the enzyme associates with the parental centrioles and deuterosomes (Hoh et al., 2012; Zhao et al., 2013). This protein is the master regulator of centriole assembly and duplication during cell division. However, recent studies found that blocking PLK4 – either with a drug or by silencing its expression – did not prevent multiciliated cells from developing the right number of centrioles and cilia, despite causing a delay in A-phase (Nanjundappa et al., 2019; Mercey et al., 2019a; Mercey et al., 2019b; Zhao et al., 2019; Wong et al., 2015). This led to the conclusion that PLK4 is dispensable for centriole amplification in epithelial progenitors. Now, in eLife, Andrew Holland and co-workers from Johns Hopkins University – including Gina LoMastro as first author – report new results that contradict this theory (LoMastro et al., 2022).

The team created mouse models which contained a modified version of the gene that encodes PLK4: in cells expressing another enzyme called Cre recombinase, the PLK4 gene no longer produced the protein, or only produced an enzymatically inactive form. LoMastro et al. found that epithelial progenitor cells lacking PLK4, or those with a non-functioning version, were unable to amplify their centrioles. Furthermore, the mutant cells could not differentiate into the multiciliated cells in the trachea or the ependymal cells lining the ventricular system in the brain, both in vitro and in vivo.

These findings provide strong evidence that PLK4 and its enzymatic activity are essential for centriole amplification in multiciliated cells. So, why did several high-quality studies appear to show the opposite result? LoMastro et al. found that when the concentration of the drug used in the previous studies was increased (from 1.5 µM to 5–10 µM), it was able to inhibit centriole amplification in ependymal and tracheal multiciliated cells. This suggests that the dose of drug needed to block A-phase is much higher than the amount required to inhibit centriole duplication in cycling cells – which makes sense given that the level of PLK4 is many times higher in A-phase than during the cell cycle (Hoh et al., 2012). Furthermore, LoMastro et al. found that multiciliated cells in the trachea have specialized proteins that pump out the inhibitor, which likely reduced the effectiveness of the drug.

Low drug doses are only part of the story. Previous studies also failed to block A-phase by silencing the expression of the gene for PLK4 using a method called RNAi, even when this was supplemented with the inhibiting drug (Nanjundappa et al., 2019; Zhao et al., 2019). It has since been discovered that the kinetics of building centrioles from scratch are highly dependent on the amount of PLK4 (Nabais et al., 2021), suggesting that cells can tell the difference between having little amounts of the enzyme and none at all. Unlike the Cre recombinase approach used by LoMastro et al., the RNAi technique does not fully eliminate gene expression and cells are likely to still have very low levels of the protein. This small amount of PLK4 may be enough to drive A-phase, which may explain why the epithelial progenitor cells in these prior experiments were still able to amplify their number of centrioles.

The drug may have failed to potentiate the effects of RNAi due to insufficient levels of the inhibitor being administered. Furthermore, this drug is known to increase PLK4 levels by preventing the protein from phosphorylating itself so it can be degraded (Nigg and Holland, 2018). Therefore, if the drug is only partially effective and inhibits some but not all PLK4 molecules, protein levels might still be able to rise, exacerbating the number of PLK4 enzymes that remain unblocked.

Further experiments are needed to investigate these possibilities. Nevertheless, it seems clear that PLK4 is the master of centriole assembly, with multiciliated cells being no exception. Several important questions remain. For instance, much remains unknown about the molecular mechanisms PLK4 activates to drive centriole assembly in cycling and multiciliated cells. In addition, it is poorly understood how the quantity of centrioles is coordinated with the size of the cell’s apical surface. LoMastro et al. found that the apical surface was smaller in PLK4 mutants that were unable to undergo centriole amplification. Since previous studies also found that expanding the apical surface leads to higher centriole numbers, this suggests that both processes are tightly coordinated (Nanjundappa et al., 2019; Kulkarni et al., 2021). Understanding how PLK4 regulates the relationship between cell shape and centriole number will be one of the next frontiers in the field.
